# Adherence to Disease and Injury Standardized Surveillance Categories in Two U.S. Africa Command Exercises, 2024

**Published:** 2026-03-06

**Authors:** Zachary. J. Rupert, Dianne N. Frankel, Michelle A. Dressner, David R. Sayers

**Affiliations:** School of Medicine, Uniformed Services University of the Health Sciences, Bethesda, MD: Lt Col Rupert; Office of the Command Surgeon, U.S. Africa Command, U.S. Department of War: Lt Col Frankel, Ms. Dressner; Department of Preventive Medicine and Biostatistics, Uniformed Services University of the Health Sciences: Lt Col Sayers

Disease and non-battle injury (DNBI) is a significant threat to military operations, historically exceeding combat injuries in deployed settings. ^1 - 4^ Disease and injury (D&I) surveillance supports health risk assessment for the purpose of instituting interventions as needed to promote and maintain the health of deployed forces. ^5 - 7^ Defense Health Agency Procedural Instruction (DHA-PI) 6490.03: Deployment Health, effective June 19, 2019, defines standardized surveillance categories for D&I reporting. ^6^ While U.S. Department of War (DOW) policy prescribes electronic systems such as the Disease Reporting System internet (DRSi) and ESSENCE, ^6^ the austere nature of expeditionary operations often necessitates reliance on paper documentation, where adherence to these guidelines has not been described.

D&I data from 2 exercises, African Lion and Flintlock, held in the U.S. Africa Command (USAFRICOM) Area of Responsibility (AOR) in 2024 were evaluated. The absolute and relative D&I burden from each exercise was calculated and compared with DHA-PI 6490.03 for category consistency and standardization. De-identified D&I surveillance data were obtained from the AFRICOM Surgeon's Office, Southern European Task Force–Africa, and Special Operations Command–Africa. D&I entries were submitted by field medical teams—comprising Guard and active duty physicians, nurse practitioners, physician assistants, and combat medics—in accordance with exercise-specific reporting requirements, primarily using paper logs and consolidated after-action reports. The project was reviewed and approved by the Institutional Review Board of the Uniformed Services University of the Health Sciences.

During the African Lion 2024 exercise (33 days), 314 D&I cases were reported within 13 categories **(Table)** . Only 8 of the 13 categories (61.5%) and 155 cases (49.4%) conformed to standardized surveillance guidelines in accordance with DHA-PI 6490.03. The Flintlock 2024 exercise (12 days) recorded 203 D&I events within 18 categories. Compared to the DHA-PI 6409.09 standardized categories, 7 of 18 categorizations (38.9%) and 113 D&I cases (55.7%) were recorded correctly. While the high relative burden of respiratory (upper) cases (23.2%) in African Lion and gastrointestinal cases (30.0%) in Flintlock suggest significant environmental threats, the use of standardized surveillance categories in only 49.4% and 55.7% of entries, respectively, limits the ability to meaningfully correlate these events with health risk assessments or location-specific risk mitigation.

**TABLE:** Case Counts and Disease and Injury Category for U.S. Africa Command African Lion and Flintlock Exercises, 2024

African Lion				Flintlock			
Disease and Injury Category	Cases		Standardized Category	Disease and Injury Category	Cases		Standardized Category
	No.	%	(x = yes)		No.	%	(x = yes)
Other medical	103	32.8		Gastrointestinal	61	30	x
Respiratory (upper)	73	23.2	x	Musculoskeletal	35	17.2	
Orthopedic	49	15.6		Ear, nose, throat (ENT)	25	12.3	
Gastrointestinal	34	10.8	x	Dermatological	20	9.9	x
Dermatological	20	6.4	x	Ophthalmological	11	5.4	x
Influenza-like illness	15	4.8	x	Dental	9	4.4	
Ophthalmological	7	2.2	x	Insect, arachnid, animal bites, stings	8	3.9	
Dental	5	1.6		Climactic injuries (heat stroke, rash)	8	3.9	x
Heat	3	1	x	Neurological	8	3.9	x
Neurological	2	0.6	x	Gynecological, urological	4	2	
Pulmonary, respiratory	1	0.3		Soft tissue	4	2	
Rash	1	0.3	x	Respiratory	3	1.5	x
Other	1	0.3		Vector-borne illness (malaria)	2	1	
				Fever, unexplained (<7 days)	1	0.5	x
				Influenza	1	0.5	
				Cardio-, chest pain	1	0.5	
				Allergy	1	0.5	
				Eye injury	1	0.5	

Abbreviations: No., number; ENT, ear, nose, throat; <, less than.

This descriptive analysis demonstrated inconsistent adherence of D&I surveillance to published military guidelines. Reporting and categorization of D&I during these exercises highlights the need for enhancing technical and administrative readiness in austere, resource-limited operational environments. While DOW electronic health records (e.g., Theater Medical Data Store) are designed to feed into standardized reporting systems, use of paper documentation in these austere environments prevents this automation.

Accurate documentation is needed for actionable medical readiness and planning. ^1 , 5^ Furthermore, lack of adherence to standardized case definitions at the point of care limits the operational value of surveillance; a list of illnesses and injuries without proper classification is ineffective for ensuring force health protection. Even in austere environments, and perhaps especially in those environments, standardized and timely data are essential for early threat detection and operational decision-making.

Recommended courses of action to combatant commands include prioritization of efforts to improve D&I surveillance by incorporating surveillance strategy into operational plans and orders (Annex Q); modifying field documentation tools (e.g., Standard Form 600, Chronological Record of Medical Care) to include D&I checkboxes; and integrating preventive medicine assets to provide just-in-time training and data quality assurance.
